# Midgut transcriptomal response of the rice leaffolder, *Cnaphalocrocis medinalis* (Guenée) to Cry1C toxin

**DOI:** 10.1371/journal.pone.0191686

**Published:** 2018-01-23

**Authors:** Yajun Yang, Hongxing Xu, Yanhui Lu, Caiyun Wang, Zhongxian Lu

**Affiliations:** State Key Laboratory Breeding Base for Zhejiang Sustainable Pest and Disease Control, Institute of Plant Protection and Microbiology, Zhejiang Academy of Agricultural Sciences, Hangzhou, China; Chinese Academy of Agricultural Sciences Institute of Plant Protection, CHINA

## Abstract

*Cnaphalocrocis medinalis* (Guenée) is one of the important insect pests in rice field. Bt agents were recommended in the *C*. *medinalis* control and Bt rice is bred as a tactic to control this insect. However, the tolerance or resistance of insect to Bt protein is a main threat to the application of Bt protein. In order to investigate the response of *C*. *medinalis* transcriptome in defending a Cry1C toxin, high-through RNA-sequencing was carried in the *C*. *medinalis* larvae treated with and without Cry1C toxin. A total of 35,586 high-quality unigenes was annotated in the transcriptome of *C*. *medinalis* midgut. The comparative analysis identified 6,966 differently expressed unigenes (DEGs) between the two treatments. GO analysis showed that these genes involved in proteolysis and extracellular region. Among these DEGs, carboxylesterase, glutathione S-transferase and P450 were differently expressed in the treated *C*. *medinalis* midgut. Furthermore, trypsin, chymotrypsin, and carboxypeptidase were identified in DEGs, and most of them up-regulated. In addition, thirteen ABC transporters were downregulated and three upregulated in Cry1C-treated *C*. *medinalis* midgut. Based on the pathway analysis, antigen processing and presentation pathway, and chronic myeloid leukemia pathway were significant in *C*. *medinalis* treated with Cry1C toxin. These results indicated that serine protease, detoxification enzymes and ABC transporter, antigen processing and presentation pathway, and chronic myeloid leukemia pathway may involved in the response of *C*. *medinalis* to Cry1C toxin. This study provides a transcriptomal foundation for the identification and functional characterization of genes involved in the toxicity of Bt Cry protein against *C*. *medinalis*, and provides potential clues to the studies on the tolerance or resistance of an agriculturally important insect pest *C*. *medinalis* to Cry1C toxin.

## Introduction

The rice leaffolder, *Cnaphalocrocis medinalis* (Guenée), is one of the important migratory insect pests of rice in China [1–2]. It widely distributes in humid tropical and temperate regions of Oceania, Africa, and Asia [3–4]. It scrapes folds and feeds the rice leaves causing the reduction of photosynthetic activity [1,5]. Heavy occurrence often happens in main rice-growing areas like China, Japan, Korea, Vietnam, Philippines, and so on [3], and frequently results in great rice yield losses [2]. The damage area by *C*. *medinalis* were more than 20 million hm^2^ in China in the most of years ranged from 2003 to 2010 [2]. A report from the Ministry of Agriculture, China showed that the damage area by *C*. *medinalis* was up to 15.5 million hm^2^ in 2015 [4].

So far, the control of *C*. *medinalis* mainly relied on the chemicals. However, the misuse and overuse of chemicals resulted in many negative issue such as insect resistance, resurgence, environmental pollution, human health concerned by public [6]. Environment-friendly methods are recommended in the pest control. *Bacillus thuringiensis*, as one of the important biological agents has been applied in the pest control for more than 60 years due to its crystal protein with insecticidal activity against insect pests [7–9]. *Cry* genes derived from *B*. *thuringiensis* were successfully transferred into plants like cotton, rice and these plants harbored resistance to Lepidopteran insect pests [10–13]. In China, Bt agent was recommended as a friendly bio-agent to use in the control of *C*. *medinalis* and Bt rice in China was approved to release for production test in limited area in 2009, but not commercially planted[11]. The application of Bt protein and Bt crop could greatly reduce the use of chemical insecticides. However, insect resistance is a big threat to the application of Bt protein and Bt crop. So far, at least eight insect pests evolved resistance against Bt crop or Bt protein [14–15]. Coincidentally, many insect pests evolved resistance to Bt proteins under selection with Bt protein in the laboratory condition [8,16]. Considering the sustainable use of the Bt protein or Bt rice, the potential of resistance evolution of *C*. *medinalis* against Bt protein will become an inescapable issue [17].

Midgut, one of the important organs in the insect body, not only is a place for food ingestion and utilization, but also a place for detoxification to xenobiotics [18]. Interaction of Bt protein and insect mainly occurred in midgut. Several reports revealed that resistance of insect against Bt protein were correlated with the variation of genes in midgut [14,19–21]. Next generation sequencing (NGS) is a strong technology which could generate large volumes of sequence information [22]. NGS provides us with unprecedented high-throughput and low-cost sequencing platforms applied in a variety of manners, including *de novo* whole-genome sequencing, resequencing of genomes to identify variations, *de novo* transcriptome and gene expression profiling, and detecting methylation patterns [23]. Now, the Illumina/Solexa sequencing technology is dominated in the NGS market, featuring high data accuracy and a broad range of applications.

In this study, transcriptome of *C*. *medinalis* midgut was sequenced and *de novo* assembled using Illumina sequencing technology. The gene expression patterns in the midgut were compared in *C*. *medinalis* larvae treated with and without Bt Cry1C protein. Moreover, the genes of digestion and detoxification system were given greater attention. The results provide clues on the roles of midgut in the response to Bt proteins, information for further study candidate genes involved in interaction of Bt protein and *C*. *medinalis* and promote the understanding of potential resistance or tolerance of *C*. *medinalis* to Bt proteins.

## Materials and methods

### Insect rearing

Adults of *C*. *medinalis* were collected with sweep net from paddy fields in suburb of Hangzhou, Zhejiang, China in August, 2015. The moths were placed in plastic cup covered with nylon mesh and fed with 10% honey solution. Eggs laid on the mesh were removed and transferred to a box with detached leaf of 45 d-old Taichung Native 1 (TN1). The larvae that hatched from eggs were cultured in the detached leaf then used in the experiment. All insect cultures were kept at 27±1°C with 70–80% RH and a photoperiod of 14: 10 (L: D) h. Insect collection is from our experimental field, and *C*. *medinalis* is not endangered or protected species.

### Treatment of *C*. *medinalis* larvae with Cry1C toxin

Fifth instar larvae of *C*. *medinalis* were selected for the feeding experiments. The larvae were reared on rice leaves dipped with Cry1C activated toxin (MP, Cavey, CWRU, US) solution which resulted in a growth inhibition but did not cause visible death of the larvae for 48 hours. Larvae treated with PBS buffer were used as negative control. All treated larvae were incubated for 48 h at 27±1°C with 70–80% RH and a photoperiod of 14: 10 (L: D) h. Approximately 150 larvae were used for each treatment.

### RNA isolation, cDNA library construction, transcriptomic sequencing

Total RNA was isolated from the midgut of *C*. *medinalis* larvae using TRIzol reagent (Invitrogen, Carlsbad, CA, USA) following the manufacturer's protocol. The total RNA quantity and purity were analyzed with Bioanalyzer 2100 and RNA 6000 Nano Lab Chip Kit (Agi-lent, CA, USA) with RIN number >7.0.

Approximately 10 μg of total RNA was subjected to isolate Poly (A) mRNA with poly-T oligo attached magnetic beads (Invitrogen). Following purification, the mRNA is fragmented into small pieces using divalent cations under elevated temperature, and the cleaved RNA fragments were reverse-transcribed to create the final cDNA library in accordance with the protocol for the mRNA-Seq sample preparation kit (Illumina, San Diego, USA). The cDNA library was sequenced run with Illumina HiSeqTM2000 sequence platform at LC Sceiences (USA) following the vendor's recommended protocols. Two or three biological replicates for each treatment were prepared.

### *De novo* RNA-seq assembly and annotation

After sequencing, the raw data were filtered against low-quantity reads and adaptor contamination using Trinity. Clean reads were *de novo* assembled using Trinity with K-mer = 25 [24]. Annotations of all the unigenes were performed by a BLASTx search against the Nr, Swiss-Prot, KOG, KEGG databases and Pfam with a cut-off E-value of ≤1e-5. We obtained the Gene Ontology terms (http://www.geneontology.org) of each *C*. *medinalis* unigene with the software Blast2GO (http://www.blast2go.org) using the default parameters [25]. Gene expression level was normalized by RSEM-based lgorithm to get RPKM value [26–27]. Based on the expression levels, thresholds of FDR<0.05 and log2 fold-change (log2FC) ≥1 were set for identifying significant differential expressed unigenes (DEGs) between control and Cry1C treated larvae.

### Enrichment analysis

All the DEGs were mapped to terms in KEGG databases to identify significantly enriched metabolic pathway or signal transduction pathways in DEGs. Pathway enrichment analysis provides all terms that significantly enriched in differentially expressed genes in comparison to the control *C*. *medinalis*. The P-value was calculated for each pathway by Fisher's exact test with a Q value threshold<0.05.

### Quantitative real-time PCR (qRT-PCR) validation

To conform the data, a subset of DEGs was validated by qRT-PCR. qRT-PCR was performed in 20 μL reaction mixtures composed of 2.5 μL of template cDNA,10 μL of 2×SYBR Green PCR Master Mix (Fermentas,Waltham, MA, USA), and 0.25 mM each primer on the CFX96 Real-Time System (Bio-Rad, Hercules, CA, USA). The selected genes were verified with the following cycling conditions: 95°C for 30 s, followed by 40 cycles of 95°C for 30 s, 60°C for 35 s. The melting curve analysis was used to analyze the specificity of the qPCR product. The sequences of the primers used are listed in S1 Table. A *ubiquinol-cytochrome c reductase* served as an internal control. The relative gene expression values were calculated using the 2^−ΔΔCt^ method [28].

## Results

### Assembly and annotation of the *C*. *medinalis* transcriptome

The transcriptomes of the midgut of *C*. *medinalis* larvae treated with and without Cry1C protein were sequenced and compared for each treatment. A total of 37.33G bases of clean reads were obtained from the cDNA libraries, and 35,586 high-quality unigenes and 65,016 transcripts were assembled (Table 1). The average gene and transcript length were 784 and 895 base pair (bp), respectively (Table 1). Mean GC% of gene and transcript were 40.66% and 39.18%, respectively. N50 (the shortest sequence length at 50% of the transcriptome) of gene and transcript were 1,211 and 1,378 bp, respectively. The size distributions of all the unigenes studied were as follows: 18,720 unigenes (17.8%) exhibited lengths of more than 2,000 bp; 17,474 unigenes (16.7%) showed lengths between 500 and 1000 bp; 26,983 unigenes (25.7%) demonstrated lengths between 300 and 500 bp; and 41,789 unigenes (39.8%) were shorter than 300 bp (S1 Fig). The size distributions of all the transcripts studied were as follows: 18,720 unigenes (17.8%) exhibited lengths of more than 2,000 bp; 17,474 unigenes (16.7%) showed lengths between 500 and 1,000 bp; 26,983 unigenes (25.7%) demonstrated lengths between 300 and 500 bp; and 41,789 unigenes (39.8%) were shorter than 300 bp (S2 Fig).

**Table 1:** Summary statistics for the transcriptome of larval midgut of *Cnaphalocrocis medinalis*.

Statistics	gene	transcript
**Total number**	35,586	65,016
**Total assembled bases**	27,902,176	58,253,881
**Min length (bp)**	201	201
**Max length (bp)**	17,428	17,428
**length > 2 kb**	2,725	6,530
**Average length (bp)**	784	895
**Median length (bp)**	471	572
**N50 length (bp)**	1,211	1,378
**Mean (G + C)s (%)**	40.66	39.18
**Median (G + C)s (%)**	38.60	36.60

To annotate these unigenes, All unigene sequences were against the protein databases (Nr, SwissProt, KEGG, COG, Pfam, GO) using BLASTx (E-value<e-5). Only 16,741 unigenes (47.04% of all unigenes) displayed Nr annotations. 9,789 unigenes (27.51% of all unigenes) displayed swiss-prot annotations. 12,317 unigenes (34.61% of all unigenes) displayed Pfam annotations. 7,401unigenes (20.80% of all unigenes) displayed KEGG annotations. 11,010 unigenes (30.94% of all unigenes) displayed KOG annotations. 9,003 unigenes (25.30% of all unigenes) displayed KEGG annotations. 11,010 unigenes (30.94% of all unigenes) displayed GO annotations. Only small partial genes were annotated to the databases because of the short nucleotide lengths and the lack of *C*. *medinalis* genomic information.

**Fig 1:**
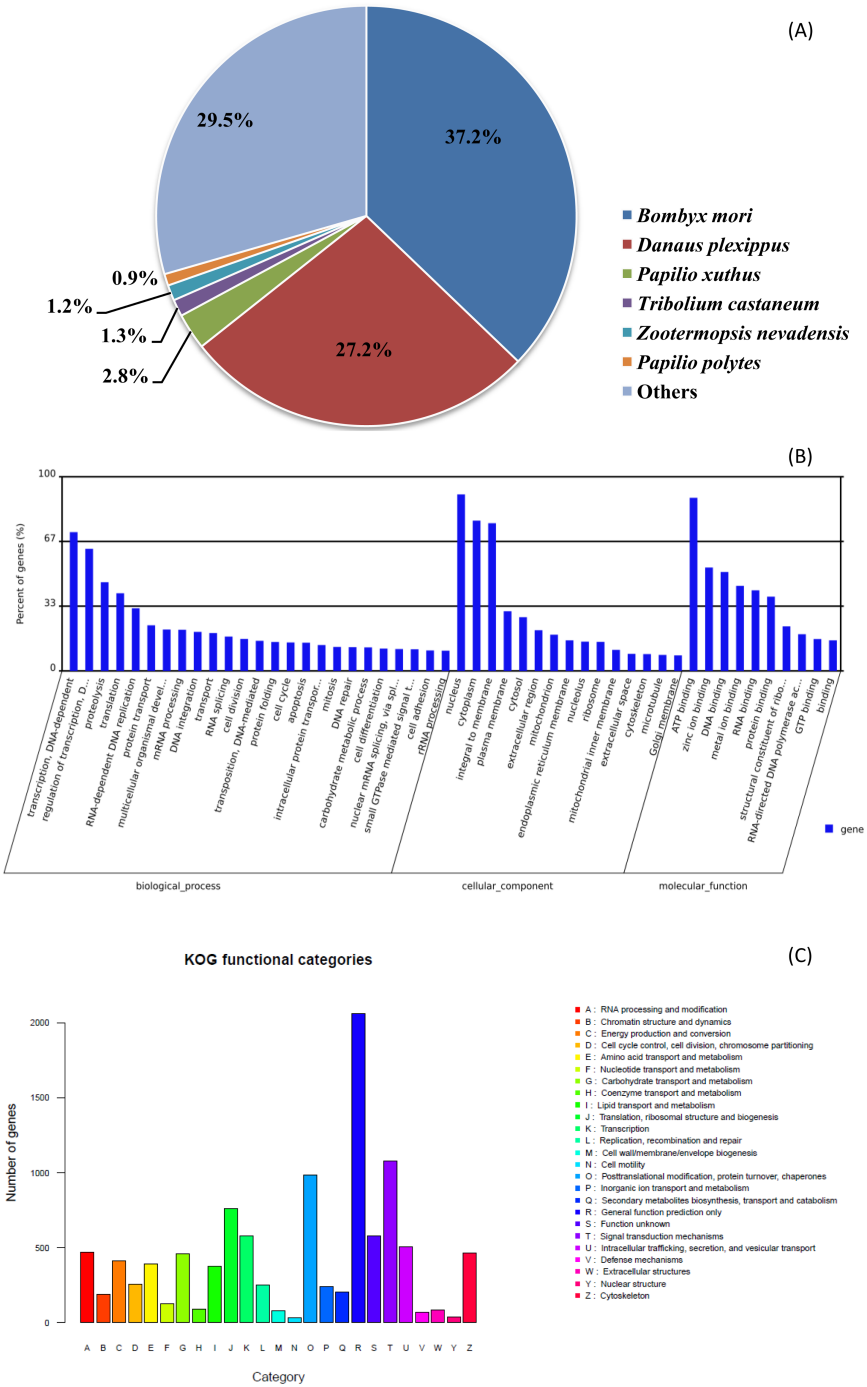
Overview of genes annotations from *Cnaphalocrocis medinalis* midgut. (A) Species distribution of the BLASTX results. (B) GO categories of all unigenes and DEGs. (C) euKaryotic orthologous Groups (KOG) classification.

Species-specific distribution revealed that 37.2% of the *C*. *medinalis* sequences matched with the silkworm, *Bombyx mori*. In addition, 27.2% of the *C*. *medinalis* sequences resembled those of *Danaus plexippus*, followed by those of *Papilio xuthus* (2.8%), *Tribolium castaneum* (1.3%), *Zootermopsis nevadensis* (1.2%), *Papilio polytes* (0.9%) (Fig 1A).

### Classifications for *C*. *medinalis* unigenes

GO assignments (level 2) were used to predict the functions of the *C*. *medinalis* unigenes. A total of 12,653 unigenes were assigned GO terms of three categories (biological process, cellular component, and molecular function) based on sequence homology. The dominant items of the three categories were nucleus, ATP-binding, cytoplasm, integral to membrane, transcription DNA-dependent. (Fig 1B).

The *C*. *medinalis* unigenes were also annotated with COG classifications. Among the 11,010 unigenes with COG annotations, 21% (2,063) belonged to the cluster of general function; followed by 10% (1,079) under the cluster of Signal transduction mechanisms; and 10% (986) under the cluster of Posttranslational modification, protein turnover, chaperones. By contrast, Cytoskeleton corresponded to the smallest clusters (Fig 1C).

The *C*. *medinalis* unigenes were also annotated with KEGG classifications. Among the 9,003 unigenes with KEGG annotations, 21% (2,145) belonged to the cluster of *Metabolism*; followed by 10% (1,014) under the cluster of *Organismal Systems*; and 10% (991) under the cluster of *Human Diseases*. *Genetic Information Processing*, *Cellular Processes*, *Environmental Information Processing*.

### Overview on complex response of *C*. *medinalis* to Cry1C toxin

**Fig 2:**
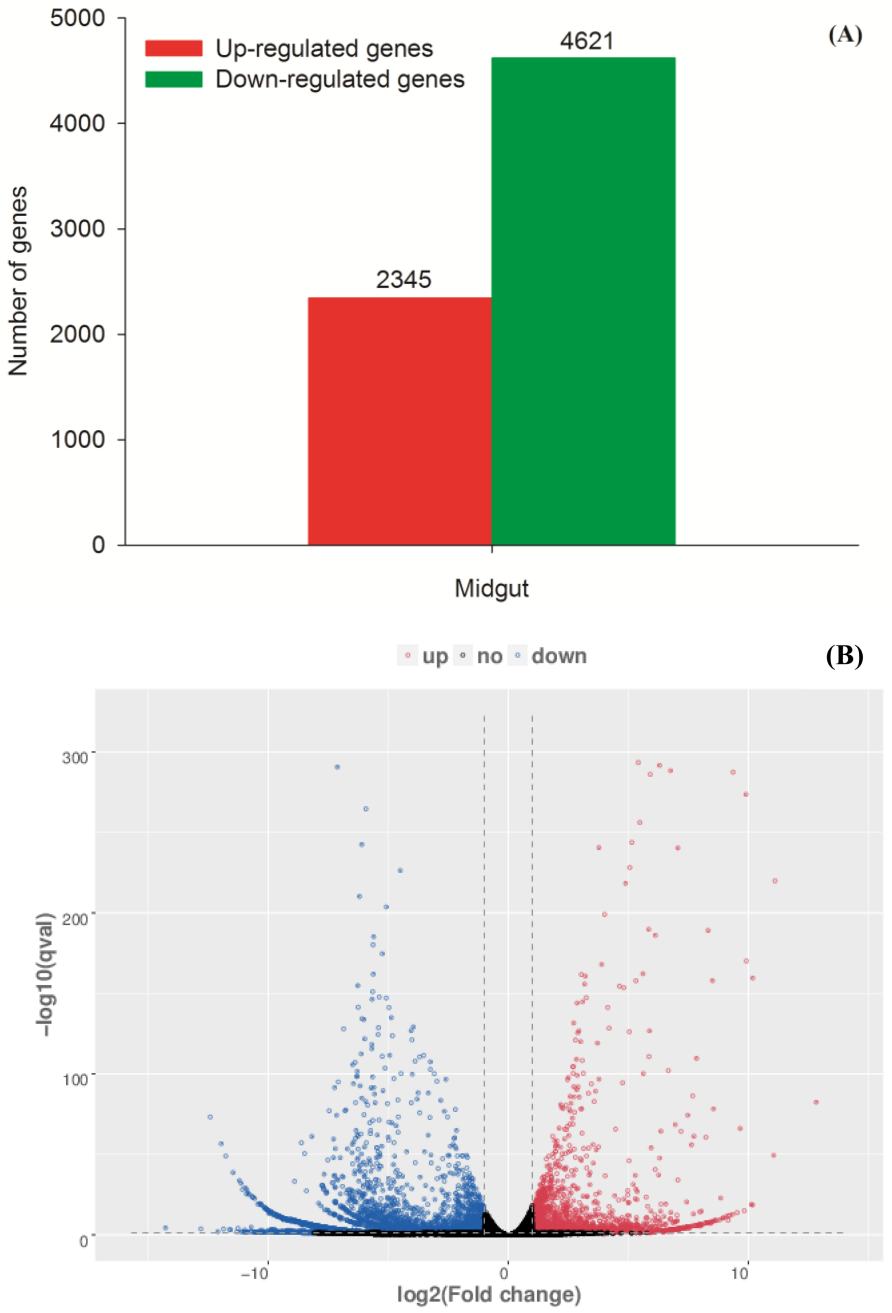
Differentially expressed unigenes (DEGs) in the midgut of *Cnaphalocrocis medinalis* larvae fed with Cry1C toxin. (A) Number of DEGs. (B) Volcano plot to show the fold change and error rates. The non-DEGs are indicated by black dots, the DEGs up-regulated are indicated by red dots, and the DEGs down-regulated are indicated by blue dots.

The number of DEGs in the midgut of *C*. *medinalis* treated with Cry1C was 6,966. Of that, 4,621 genes were down-regulated, and 2,345 genes were up-regulated (Fig 2A). The changed ratios of most DEGs accumulated between 2^−15^ and 2^13^ (Fig 2B). For the top 20 upregulated unigenes in the midgut of *C*. *medinalis* treated with Cry1C, three unigenes had explicit annotations, i.e., adenosinetriphosphatase, nucleoprotein TPR, and heat shock 70kDa protein 1/8 (S2 Table). Meanwhile, for the top 20 downregulated unigenes in the midgut of *C*. *medinalis* treated with Cry1C, 10 unigenes had explicit annotations, i.e., encoded NADH dehydrogenase I subunit 4, beta-2-microglobulin, glypican 5, CD74 antigen, tyrosine 3-monooxygenase/tryptophan 5-monooxygenase activation protein, valyl-tRNA synthetase, tyrosine 3-monooxygenase/tryptophan 5-monooxygenase activation protein, valyl-tRNA synthetase, protein transport protein SEC61 subunit alpha, saposin (S3 Table).

**Fig 3:**
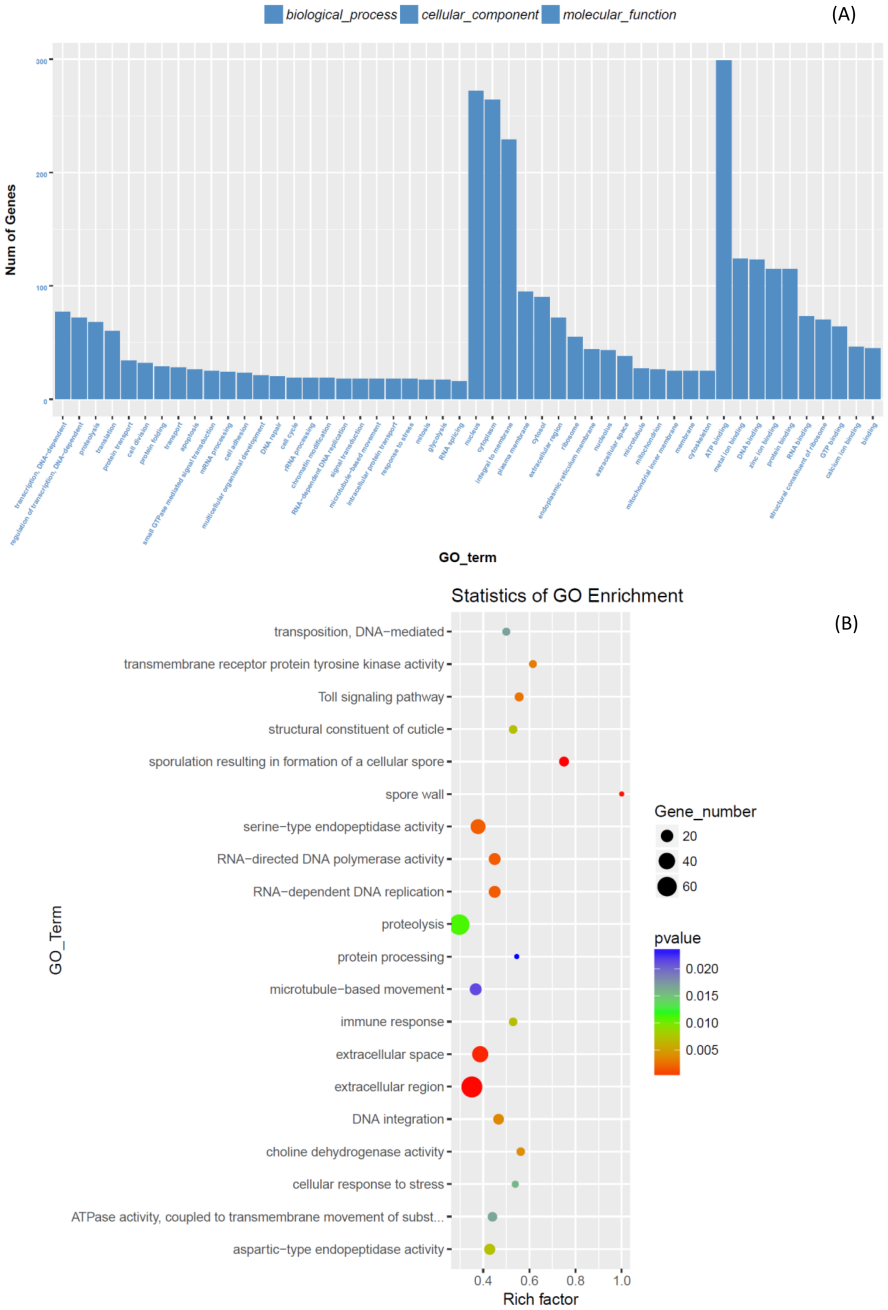
Statistic of GO enrichments of DGEs. (A) Histogram. (B) Scatter plot.

From the statistic of GO enrichment, most of DEGs involved in transcription DNA-dependent, regulation of transcription, and proteolysis under biological process; most of DEGs involved in nucleusm, cytoplasm, and integral to membrane under cellular component function item; most of DEGs involved in ATP-binding, metal ion binding, and DNA binding under molecular function (Fig 3A). According to the GO enrichment scatterplot (Fig 3B), large amount of genes involved in proteolysis, extracellular region, extracellular space, and serine-type endopeptidase activity were differently expressed in larval midgut of *C*. *medinalis* treat with and without Cry1C toxin.

To validate the transcriptome data, qRT-PCR analysis was performed for DGEs (S1 Table). The qRT-PCR validation results were presented as fold-changes normalized to the *ubiquinol-cytochrome c reductase* gene. A comparison of the mean values of qRT-PCR results and the DEGs is summarized in S3 Fig. The consistent expression trend in qRT-PCR results and the original DEGs indicates the reliability of gene expression profiling from transcriptome analysis.

### Interest genes and Bt related genes in the transcriptome of *C*. *medinalis* midgut

**Fig 4:**
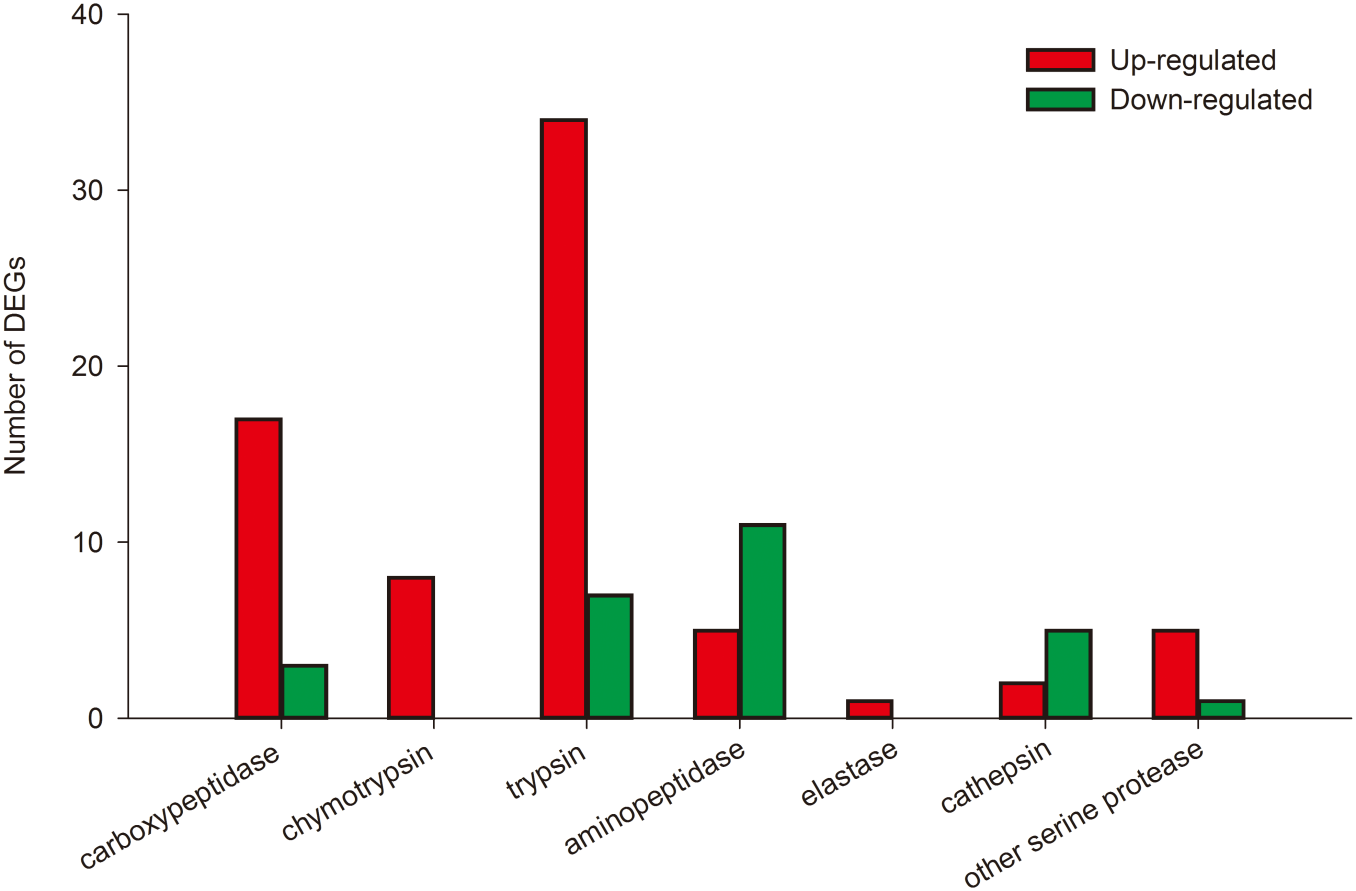
Serine proteases differently expressed in *Cnaphalocrocis medinalis* larvae treated with Cry1C toxin.

**Fig 5:**
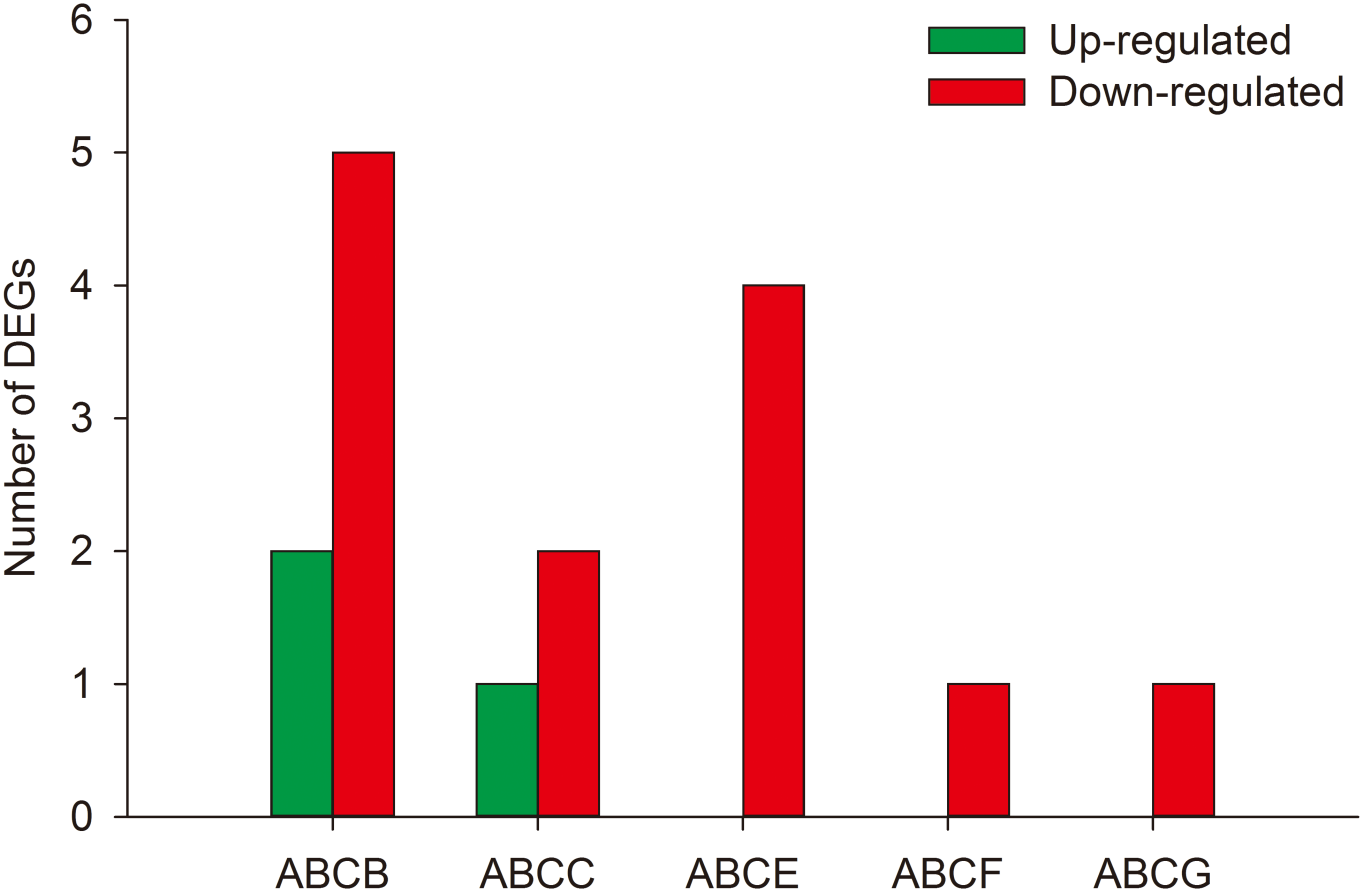
ABC transporters differently expressed in *Cnaphalocrocis medinalis* larvae treated with Cry1C toxin.

Here, we investigated DEGs potentially involved in insecticidal tolerance/resistance in *C*. *medinalis*, including metabolic and Bt-related genes. DEGs sequences encoding enzymes function ingestion and detoxification and receptors of the insecticide and toxins were extracted from the RNA-seq database from the *C*. *medinalis* midgut. Here, alkaline phosphatase, cytochrome P450, glutathione S-transferase (GST), carboxylesterase, ABC transporter, and serine protease including transmembrane protease, trypsin and chymotrypsin were (FDR < 0.05 and the absolute value of log2FC ≥ 1) were found significant different in the midgut of *C*. *medinalis* larvae treated with Cry1C protein (Table 2). A total of thirteen DEGs encoding carboxylesterases were differentially expressed between treated and untreated *C*. *medinalis*, of which 11 of these unigenes were up-regulated and two were down-regulated in the Cry1C-treated *C*. *medinalis* (S4 Table). A total of 99 serine protease genes were differently expressed including trypsin, chymotrypsin, carboxypeptidase, aminopeptidase, elastase, cathepsin and so on (Fig 4). Fourty-one unigenes encoding trypsin were differentially expressed between treated and untreated *C*. *medinalis*, of which thirty-four unigenes were up-regulated and seven were down-regulated in the Cry1C-treated *C*. *medinalis* (Table 3). Eight chymotrypsin were 1.24- to 3.69- fold upregulated. Twenty carboxypeptidase genes were differently expressed with 17 genes upregulated and 3 downregulated in the Cry1C-treated *C*. *medinalis* (Table 4). Four aminopeptidase N were upregulated and two downregulated, and two alkaline phosphatase were upregulated in the midgut of *C*. *medinalis* larvae treated with Cry1C. One cadherin in Cry1C treated *C*. *medinalis* larvae were differently downregulated by 1.36-fold compared with those of the untreated larvae. The DEGs were annotated to the ABC transporter including five subfamilies (ABCB, ABCC, ABCE, ABCF and ABCG), and three upregulated and 13 down regulated (Fig 5).

**Table 2:** Differently expressed unigenes potentially involved in *Cnaphalocrocis medinalis* response to Cry1C toxin.

Genes	Treated/Control ^a^		
	up	down	Total
**Metabolic insecticide resistance and insecticide targets**			
**Cytochrome P450 monooxygenase**	3	3	6
**Carboxylesterase**	11	2	13
**Glutathione S-transferase**	2	1	3
**Acetylcholinesterase**	0	0	0
**Nicotinic acetylcholine receptor**	0	0	0
**GABA receptor**	0	1	1
**Neuropeptide receptor**	0	2	2
**Glutamate receptor**	0	1	1
**G-protein coupled receptor**	0	0	0
**Ryanodine receptor**	0	2	2
**Lipophorin receptor**	0	0	0
**Sodium channel**	0	0	0
**Chloride channel**	0	1	1
**NADH dehydrogenase**	0	9	9
**NADH oxidoreductase**	0	0	0
**Catalase**	0	1	3
**Peroxidase**	2	9	11
**Superoxide dismutase**	1	3	4
**Bt resistance**			
**Cadherin**	0	1	1
**Aminopeptidases N**	4	2	6
**Alkaline phosphatase**	2	0	2
**Glycolipid**	0	0	0
**ABC transporter**	3	13	16
**carboxypeptidase**	17	3	20
**trypsin**	34	7	41
**chymotrypsin**	8	0	8
**cathepsin**	2	5	7

^a^ Differentially expressed genes listed here have a FDR of <0.05 and the absolute value of log2ratio ≥ 1 between *C*. *medinalis* larvae treated with and without Cry1C toxin.

**Table 3:** Trypsin genes of *Cnaphalocrocis medinalis* midgut in response to the ingestion of Cry1C toxin.

Gene ID	Annotation	Treated vs Control^a^		
		Log2FC	FDR	regulated
**comp57649_c2**	trypsin	2.72	2.19E-132	up
**comp53936_c0**	trypsin	2.89	2.53E-126	up
**comp58709_c0**	trypsin	2.57	8.96E-87	up
**comp62679_c0**	trypsin	2.61	1.72E-82	up
**comp52245_c0**	trypsin	2.40	2.94E-81	up
**comp52333_c0**	trypsin	3.05	3.82E-79	up
**comp59607_c0**	trypsin	3.02	4.98E-79	up
**comp53824_c0**	trypsin	2.57	1.17E-77	up
**comp55688_c0**	trypsin	2.55	2.58E-56	up
**comp50084_c0**	trypsin	3.37	6.08E-38	up
**comp58071_c0**	trypsin	1.81	1.19E-36	up
**comp59543_c0**	trypsin	2.28	4.09E-34	up
**comp55740_c0**	trypsin	1.92	6.33E-33	up
**comp59573_c0**	trypsin	1.48	1.99E-29	up
**comp51336_c0**	trypsin	3.79	5.20E-27	up
**comp54289_c0**	trypsin	1.79	2.94E-26	up
**comp59285_c0**	trypsin	2.24	2.88E-21	up
**comp50389_c0**	trypsin	4.20	2.35E-18	up
**comp53422_c0**	trypsin	2.46	1.35E-16	up
**comp55884_c0**	trypsin	2.28	3.08E-16	up
**comp51937_c0**	trypsin	2.80	4.03E-16	up
**comp46739_c0**	trypsin	3.24	2.46E-12	up
**comp43712_c0**	trypsin	3.81	2.48E-09	up
**comp53870_c1**	trypsin	1.61	6.66E-09	up
**comp61336_c0**	trypsin	1.15	7.34E-07	up
**comp47248_c0**	trypsin	2.57	3.22E-06	up
**comp53870_c0**	trypsin	2.61	7.99E-05	up
**comp46414_c0**	trypsin	2.62	2.28E-03	up
**comp51898_c0**	trypsin	1.67	4.34E-03	up
**comp40870_c1**	trypsin	2.36	5.12E-03	up
**comp53728_c0**	trypsin	1.44	1.33E-02	up
**comp40870_c0**	trypsin	2.95	1.70E-02	up
**comp56917_c0**	trypsin	1.18	4.07E-02	up
**comp47218_c0**	trypsin	1.72	4.76E-02	up
**comp62257_c0**	trypsin	-1.05	6.09E-15	down
**comp62572_c0**	trypsin	-1.40	1.11E-09	down
**comp50375_c0**	trypsin	-4.12	1.04E-05	down
**comp59978_c0**	trypsin	-1.05	2.51E-04	down
**comp59413_c0**	trypsin	-1.73	1.03E-03	down
**comp46878_c0**	trypsin	-10.52	1.57E-02	down
**comp58010_c0**	trypsin	-1.24	4.26E-02	down

^a^ Differentially expressed genes listed here have a FDR of <0.05 and the absolute value of log2ratio ≥ 1 between *C*. *medinalis* larvae treated with and without Cry1C toxin.

**Table 4:** Carboxypeptidase genes of *Cnaphalocrocis medinalis* midgut in response to the ingestion of Cry1C toxin.

Gene ID	Annotation	Treated vs Control^a^		
		Log2FC	FDR	regulated
comp58247_c0	carboxypeptidase	1.42	1.13E-05	up
comp62619_c0	carboxypeptidase A	2.36	3.14E-51	up
comp61191_c0	carboxypeptidase A	2.19	2.05E-18	up
comp55473_c0	carboxypeptidase A	2.38	4.30E-12	up
comp58294_c0	carboxypeptidase A	1.23	2.09E-11	up
comp60636_c0	carboxypeptidase A	1.51	6.93E-10	up
comp57980_c0	carboxypeptidase A	2.07	4.13E-08	up
comp61570_c0	carboxypeptidase A	1.08	2.82E-07	up
comp58731_c0	carboxypeptidase A	1.54	1.08E-05	up
comp57828_c2	carboxypeptidase A	1.08	4.94 E-04	up
comp51659_c1	carboxypeptidase A	1.99	6.15 E-03	up
comp57845_c0	carboxypeptidase A	1.18	2.81E-02	up
comp55631_c0	carboxypeptidase A2	3.13	2.12E-54	up
comp60005_c0	carboxypeptidase A2	2.68	3.70E-31	up
comp59859_c0	carboxypeptidase A2	3.06	1.52E-14	up
comp58033_c0	carboxypeptidase A4	5.14	1.32E-244	up
comp61876_c0	lysosomal Pro-X carboxypeptidase	1.63	5.50E-13	up
comp54790_c0	carboxypeptidase A	-3.54	4.03E-14	down
comp45043_c0	carboxypeptidase A	-4.51	1.57E-03	down
comp62420_c0	lysosomal Pro-X carboxypeptidase	-1.87	1.02E-07	down

^a^ Differentially expressed genes listed here have a FDR of <0.05 and the absolute value of log2ratio ≥ 1 between *C*. *medinalis* larvae treated with and without Cry1C toxin.

### Significant enriched pathways in the *C*. *medinalis* larvae treated with Cry1C toxin

Metabolic pathway enrichment analysis demonstrated that 770 DEGs involved in 232 pathways (S5 Table) might play a role in the response of *C*. *medinalis* to Cry1C toxin. DEGs were significantly enriched in two functional categories (antigen processing and presentation, and chronic myeloid leukemia) in *C*. *medinalis* treated with Cry1C (Q-value < 0.05) (Table 5). Twenty-five DGEs were found in antigen processing and presentation pathway, and twelve DGEs were found in chronic myeloid leukemia pathway.

**Table 5:** Enriched pathway of DEGs in *Cnaphalocrocis medinalis* response to Cry1C toxin (Q-value<0.05).

Pathway_ID	Pathway Name	Gene number	Q-value
**ko04612**	Antigen processing and presentation	25	0.0105
**ko05220**	Chronic myeloid leukemia	12	0.0487

## Discussion

Despite *C*. *medinalis* is one of the important insect pests in rice field of Asia, and also the target insect of Bt agent and Bt rice [3,11]. But the interaction between this insect pest and Bt toxin is unclear. The response of *C*. *medinalis* to Bt toxin related with the tolerance and resistance to Bt toxin. In this study, larval midgut transcriptome was sequenced and analyzed, 35,586 high-quality unigenes were obtained, and 6,966 DEGs were found between *C*. *medinalis* larvae fed with and without Cry1C toxin. In *Plutella xylostella* midgut transcriptome, 2,925 and 2,967 unigenes were differently expressed in two resistant strains compared with susceptible strains [29]. In a pink bollworm (*Pectinophora gossipiella*) larval midgut transcriptome, 39,874 unigenes were obtained from18,623,508 assembled reads [30]. The number of bases in *C*. *medinalis* midugt transcriptome in this study was smaller than those in *P*. *xylostella* and *P*. *gossipiella* larval midgut transcriptome. In the transcriptome of the *Lymantria dispar* (gypsy moth) larval midgut in response to infection by *B*. *thuringiensis*, the DEGs primarily associated with digestive function (eg. a-amylase, lipase and carboxypeptidase), immune response (eg. C-type lectin 4), developmental genes (eg. arylphorin) as well as a variety of binding proteins including cellular retinoic acid binding protein (lipid-binding), insulin-related peptide binding protein (protein binding) and ovary C/EBPg transcription factor (nucleic acid-binding) [31]. Many DGEs involved in the metabolic pathway, ABC transporter pathway, aminopeptidases and cadherins were found between Cry1Ac-resistant and susceptible strains of *P*. *xylostella* [29]. Here we focused on the interest genes involved insecticides and Bt resistance or tolerance. These findings may facilitate the understanding of interaction of insect and Bt protein.

### Detoxification enzymes

Carboxylesterase, cytochrome P450, and glutathione S-transferase (GST) are important enzymes in insect system and function on the metabolic and detoxification of the xenobiotics. GST in *C*. *medinalis* could involved in the detoxification of chlorpyrifos [32]. Chen et al. [33] discovered that two P450 genes *CYP6CV1* and *CYP9A38* may be involved in detoxification of rice phytochemicals. Veegala and Vemuri [34] reported carboxylesterase and GST mediated in the resistance of *C*. *medinalis* to insecticide. In this study, 6 cytochrome P450, 3 GSTs, 13 carboxylesterase were significant different expression in *C*. *medinalis* treated with Cry1C, suggesting that detoxification enzymes may be involved the early response of *C*. *medinalis* larvae to Cry1C toxin. Similarly, Guo et al. [35] discovered that carboxylesterase were increased in *S*. *exigua* as the increased time of exposure to Bt cotton and considered that the allocation of carboxylesterase in body is a metabolic tolerance of herbivorous insects response to a toxic protein.

### Protect enzymes

Catalase (CAT), peroxidase (POD), and superoxide dismutase (SOD) are the important protection enzymes in insect [36]. Here, we found 1 CAT, 11 PODs and 4 SODs were found. In midgut of *C*. *medinalis* treated with Cry1C, 1 CAT, 9 PODs and 3 SODs were down regulated, and 2 other PODs and one SOD were upregulated. These results showed Cry1C impose environmental stress to *C*. *medinalis* larvae, anyhow the upregulated PODs indicated that the body of *C*. *medinalis* adjust the physiological activity to reply to Cry1C. Many reports indicated that the increase of POD is the emergence measures of the insect reply to Bt toxin [35,37].

### Serine protease

Serine proteases always function on the digestion and utilization of food, and the detoxification of exogenous proteins. Trypsin and chymotrypsin are two kinds of serine proteases and involved in the activation and degradation of Bt toxin [38–40]. The changes of serine proteases may be correlated with the resistance of the insect to Bt toxins. Tetreau et al. [41] reported an increase in larval gut proteolytic activities in a Bt resistance strain of the Dengue fever mosquito. Elleuch et al. [42] found that early degradation of Cry toxins by proteases in mosquito larvae midgut may be one of the reasons why Bt protein toxicity decreased. In our study, 34 trypsin and 8 chymotrypsin were upregulated and 7 other trypsin down-regulated. In addition, twenty carboxypeptidases were differently expressed with 17 genes up-regulated and 3 genes down-regulated. These results indicated that the trypsin, chymotrypsin and carboxypeptidases could play an important role on the tolerance of *C*. *medinalis* to Cry1C through degradation.

### Bt receptors

In the Bt action, Bt receptors are important on the formation of toxicity [19]. So far, at least cadherin, alkaline phosphatase, and aminopeptidase N (APN) were putative to be the receptor of Bt toxins in insects [15,19]. However, there are no reports on these proteins as Bt receptors in *C*. *medinalis*. Unigenes on these proteins could provide invaluable information for the further study to prove these proteins as Bt receptors in *C*. *medinalis*. A previous study in the cabbage looper (*Trichoplusia ni*) indicated that the Cry1Ac resistance was correlated with the concurrent up-regulation of APN6 and down-regulation of APN1 [43]. In our study, four APN were up-regulated (1.68- to 2.54-fold) and two APNs downregulated (2.76- and 5.57- fold) in the *C*. *medinalis* treated with Cry1C toxin. The detailed role of these APNs on the tolerance or resistance of *C*. *medinalis* to Cry1C remained to be determined. Alkaline phosphatases have also been identified as Bt receptors in many insects [19,44–46]. Chen et al. [47] found a toxin-binding alkaline phosphatase fragment could synergize Bt toxin Cry1Ac against susceptible and resistant *Helicoverpa armigera*. Here, two alkaline phosphatases were upregulated in *C*. *medinalis* larvae treated with Cry1C. According to the role of alkaline phosphatase in Bt action, alkaline phosphatases in tolerant or resistant insects to Bt toxin may do not increase, the results in our studies may indicate these two alkaline phosphatases may be not the receptor of the Cry1C or the results may be unexpected. Flores-Escobar et al. [48] found alkaline phosphatase is more important than APN for Cry1Ab toxicity, while Cry1Ac relied principally on APN1 through RNAi experiment. Cadherin is an important Bt receptor and its mutation was correlated with the insect resistance to Bt toxins [14,19,49]. Here, one unigene annotated as cadherin was downregulated in *C*. *medinalis* larvae treated with Cry1C. However, the upregulated cadherin belongs to flamingo-cadherin which is different with the cadherin in other insects related with Bt action. Additionally, the other cadherin in *C*. *medinalis* was not differently expressed. Whether this flamingo-cadherin correlated with Cry1C action remains to be determined.

### ABC transporters

ATP-binding cassette (ABC) transporters is a super family involved in various functions including the transport of lipids, inorganic ions and especially the detoxification of xenobiotics [50]. ABC transporter plays important role in drug resistance [51]. And in insect ABC transporters were shown functions in glucoside sequestration [52], the transport of eye color pigments [53] and insecticidal resistance [54]. In this study, sixteen ABC transporters attributed to 5 subfamilies were regulated in *C*. *medinalis* treated with Cry1C and 3 upregulated and 13 down regulated. Previous studies have linked ABC transporters with Cry1 resistance in seven lepidopterans including *Heliothis virescens*, *H*. *armigera*, *P*. *xylostella*, *T*. *ni*, *Ostrinia nubilalis*, *H*. *punctigera* and *B*. *mori* [29,55–59]. In the transcriptome of *P*. *xylostella* Cry1Ac-susceptible and resistant strains, many DGEs of ABC transporters were observed [29]. The role of the ABC transporters found in this study on tolerance or resistance of *C*. *medinalis* to Cry1C warrants further investigation.

### Enriched pathway

Pathway analysis indicated that two pathways were significant enriched in the *C*. *medinalis* treated with Cry1C. The antigen processing and presentation pathway had 25 DEGs in the *C*. *medinalis* treated with Cry1C. Antigen processing is an immunological process that prepares antigens for presentation to special cells of the immune system called T lymphocytes [60]. Subsequent presentation of the antigens on class I or class II major histocompatibility complex (MHC) molecules is dependent on the pathway, and MHC I and MHC II antigen presentation typically involves the endogenous and exogenous pathway of antigen processing, respectively [60]. The transporter associated with antigen processing (TAP) represents a key machinery in MHC class I antigen presentation by translocating proteasomal degradation products into the lumen of the endoplasmic reticulum (ER) for loading onto MHC class I molecules, and TAP is a member of the ABC transporters [61]. The ABC transporter pathway was an important enriched one due to previous report that ABC transporter was correlated with Bt resistance in insects [62–63]. Chronic myeloid leukemia (CML) pathway correlated with the apoptosis, and twelve DGEs were found in this pathway. ABC transporter also was important in the CML pathway [64]. In addition, a mechanism of Bt action was reported to be related to apoptosis [65–67]. These two pathways may provide more information on the Bt action and insect response to Bt protein.

In conclusion, multiple aspects were involved in the *C*. *medinalis* response to Cry1C protein from the transcriptomal data. Serine protease, detoxification enzymes, ABC transporter, antigen processing and presentation pathway, and chronic myeloid leukemia pathway may be main elements involved in the response of *C*. *medinalis* to Cry1C toxin.

## Supporting information

S1 Fig Distribution of unigenes from the midgut transcriptome of *Cnaphalocrocis medinalis*. (TIF)

S2 Fig Distribution of transcripts from the midgut transcriptome of *Cnaphalocrocis medinalis*. (TIF)

S3 Fig Validation of transcriptome data by qRT-PCR. The means of at least three biological replicates are presented as log_2_FC ± SE.
(TIF)

S1 Table List of primers of genes for the validation of transcriptome data by qRT-PCR. (DOCX)

S2 Table Top 20 downregulated unigenes in the midgut of *Cnaphalocrocis medinalis* treated with Cry1C toxin. (DOCX)

S3 Table Top 20 upregulated unigenes in the midgut of *Cnaphalocrocis medinalis* treated with Cry1C toxin. (DOCX)

S4 Table Carboxylesterase genes differently expressed in *Cnaphalocrocis medinalis* larvae treated with Cry1C toxin. (DOCX)

S5 Table KEGG summary of *Cnaphalocrocis medinalis* response to Cry1C toxin. (XLSX)
