# Supplementary figures and images for: Midgut transcriptomal response of the rice leaffolder, Cnaphalocrocis medinalis (Guenée) to Cry1C toxin
Source: PLoS One. 2018 Jan 23;13(1):e0191686. doi: 10.1371/journal.pone.0191686 (PMC5779695; doi:10.1371/journal.pone.0191686)

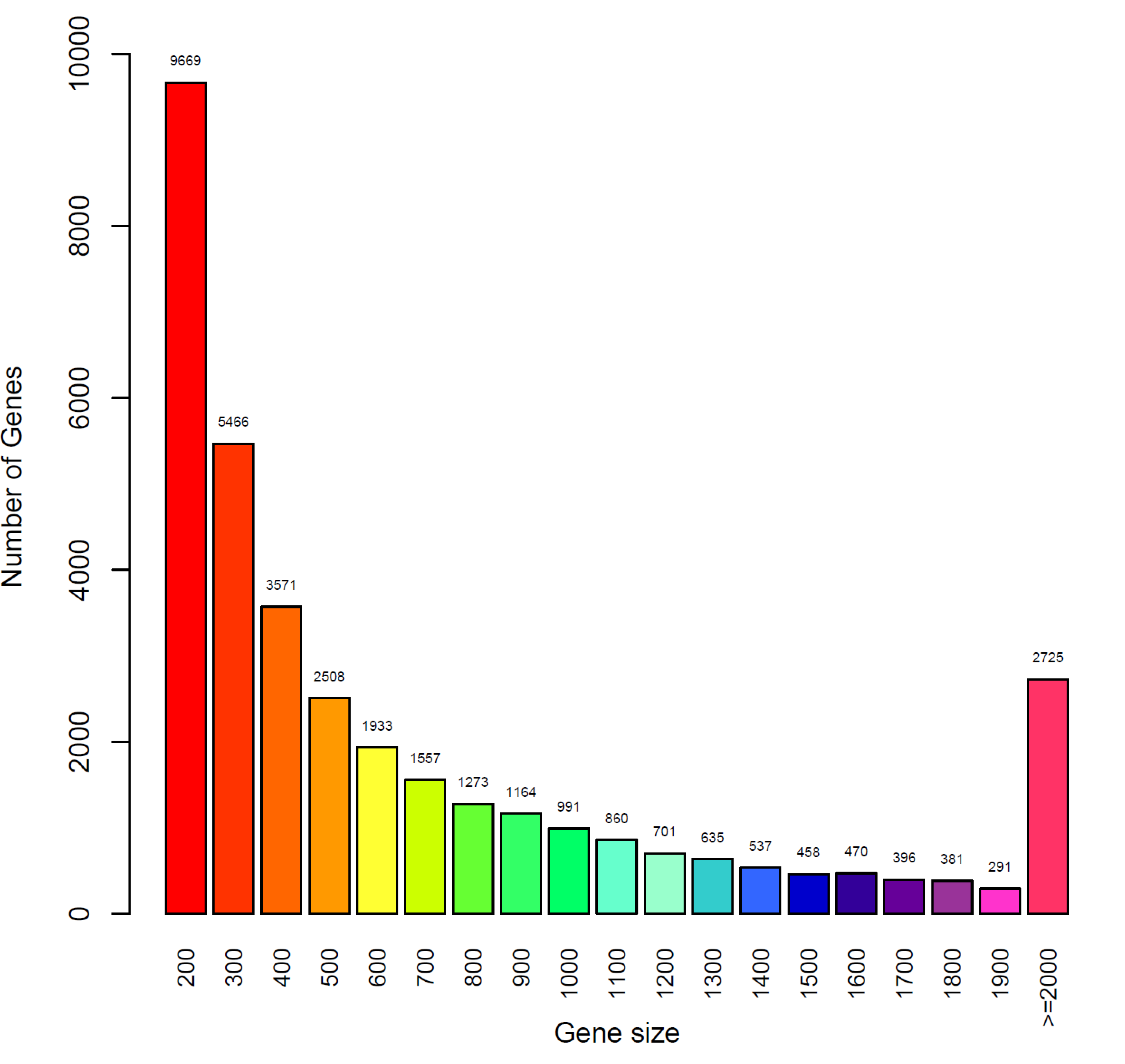

Supplement: S1 Fig — (TIF) [file pone.0191686.s001.tif]

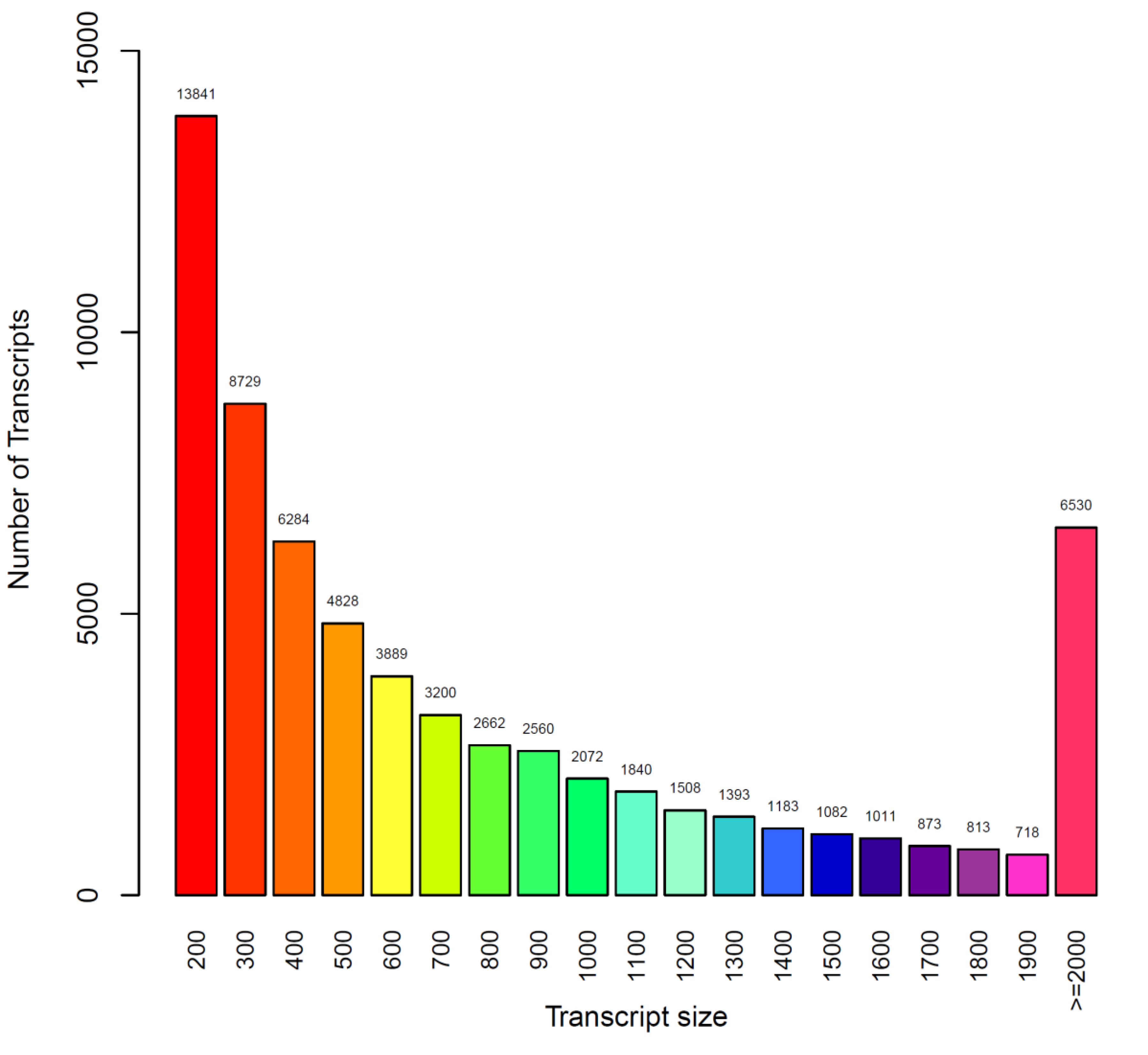

Supplement: S2 Fig — (TIF) [file pone.0191686.s002.tif]

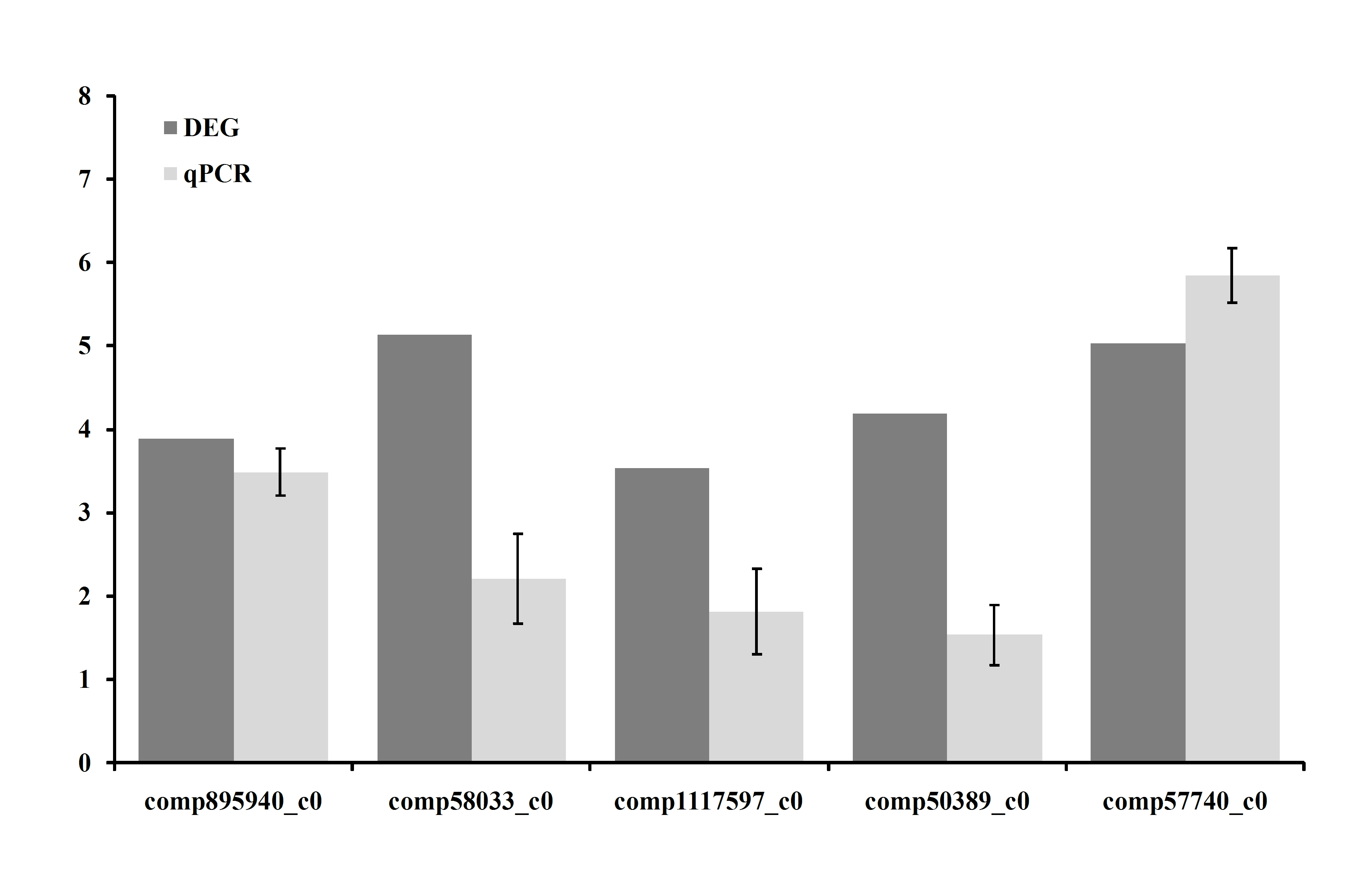

Supplement: S3 Fig — The means of at least three biological replicates are presented as log2FC ± SE. (TIF) [file pone.0191686.s003.TIF]
