# Supplementary material for: Midgut transcriptomal response of the rice leaffolder, Cnaphalocrocis medinalis (Guenée) to Cry1C toxin
Source: PLoS One. 2018 Jan 23;13(1):e0191686. doi: 10.1371/journal.pone.0191686 (PMC5779695; doi:10.1371/journal.pone.0191686)
Supplement: S1 Table — (DOCX) [file pone.0191686.s004.docx]

**S1 Table**

List of primers of genes for the validation of transcriptome data by qRT-PCR.

| Gene | Gene ID | Forward primer | Reverse primer |
| --- | --- | --- | --- |
| *carboxylesterase* | comp895940_c0 | ACTCGGGTGCAAATGTCTGGT | GGCGCCATGAACCTCTACAAA |
| *carboxypeptidase A4* | comp58033_c0 | AATTACGCGCGATGTTGGCT | CCGGCTTGCTCAATGAACCAA |
| *cytochrome P450* | comp1117597_c0 | TGAAGTTGATGGCGGCGTTT | ACTTAAAAGCGGTGGTCCTGGA |
| *trypsin* | comp50389_c0 | TGTAGAGTCGCTGGTTGTGGT | TATTCGCCCAGTTTGCTTGCC |
| *peroxidase* | comp57740_c0 | TGACATGCTGCGACAACAACG | TTGGGCGGAATCAAGATGGGT |
| *ubiquinol-cytochrome c reductase* | reference gene | ACAGTCGCCTTCAAAGCTGGT | CCAATCTGTGCCAACTTGCGT |
