# Supplementary material for: Midgut transcriptomal response of the rice leaffolder, Cnaphalocrocis medinalis (Guenée) to Cry1C toxin
Source: PLoS One. 2018 Jan 23;13(1):e0191686. doi: 10.1371/journal.pone.0191686 (PMC5779695; doi:10.1371/journal.pone.0191686)
Supplement: S2 Table — (DOCX) [file pone.0191686.s005.docx]

**S2 Table**

Top 20 upregulated unigenes in the midgut of *Cnaphalocrocis medinalis* treated with Cry1C toxin

| Gene ID | Annotation | Log2FC^a^ | FDR |
| --- | --- | --- | --- |
| comp45182_c0 | NA | 12.82 | 3.35E-83 |
| comp71829_c0 | NA | 11.11 | 1.15E-220 |
| comp45875_c0 | NA | 11.10 | 0 |
| comp58759_c0 | adenosinetriphosphatase | 11.05 | 3.50E-50 |
| comp53264_c0 | NA | 10.18 | 2.54E-160 |
| comp30793_c0 | NA | 10.17 | 2.31E-19 |
| comp55828_c0 | NA | 10.12 | 1.20E-19 |
| comp69139_c0 | nucleoprotein TPR | 9.91 | 5.96E-171 |
| comp30565_c0 | heat shock 70kDa protein 1/8 | 9.89 | 2.22E-274 |
| comp16433_c0 | NA | 9.83 | 9.61E-16 |
| comp47848_c0 | NA | 9.66 | 7.30E-67 |
| comp11767_c0 | NA | 9.53 | 1.40E-14 |
| comp70390_c0 | NA | 9.36 | 2.94E-288 |
| comp791_c0 | NA | 9.33 | 1.74E-13 |
| comp11684_c0 | NA | 9.20 | 1.13E-12 |
| comp274060_c0 | NA | 9.18 | 1.30E-12 |
| comp267150_c0 | NA | 9.15 | 7.38E-10 |
| comp520945_c0 | NA | 9.07 | 4.54E-12 |
| comp481769_c0 | NA | 8.95 | 2.69E-11 |
| comp420005_c0 | NA | 8.94 | 3.70E-11 |

^a^ Fold change was calculated as the number of reads per kilo bases per million (RPKM) of the midgut sample of *C. medinalis* treated with Cry1C divided by the RPKM of the midgut sample of *C. medinalis* treated without Cry1C.
