# Supplementary material for: Midgut transcriptomal response of the rice leaffolder, Cnaphalocrocis medinalis (Guenée) to Cry1C toxin
Source: PLoS One. 2018 Jan 23;13(1):e0191686. doi: 10.1371/journal.pone.0191686 (PMC5779695; doi:10.1371/journal.pone.0191686)
Supplement: S3 Table — (DOCX) [file pone.0191686.s006.docx]

**S3** **Table**

Top 20 downregulated unigenes in the midgut of *Cnaphalocrocis medinalis* treated with Cry1C toxin

| Gene ID | Annotation | Log2FC^a^ | FDR |
| --- | --- | --- | --- |
| comp57500_c0 | NADH dehydrogenase I subunit 4 | -14.28 | 4.07E-5 |
| comp44314_c0 | beta-2-microglobulin | -12.81 | 1.64E-4 |
| comp88054_c0 | NA | -12.42 | 5.62E-74 |
| comp52448_c0 | NA | -12.11 | 9.35E-3 |
| comp53360_c0 | NA | -11.97 | 2.01E-57 |
| comp62376_c0 | glypican 5 | -11.87 | 2.28E-4 |
| comp67604_c0 | NA | -11.78 | 8.63E-50 |
| comp118634_c0 | CD74 antigen | -11.61 | 4.70E-4 |
| comp30628_c0 | NA | -11.57 | 7.88E-4 |
| comp56761_c0 | NA | -11.47 | 1.87E-39 |
| comp268976_c0 | NA | -11.31 | 1.18E-2 |
| comp31382_c0 | NA | -11.27 | 7.71E-5 |
| comp51328_c0 | tyrosine 3-monooxygenase/tryptophan 5-monooxygenase activation protein | -11.21 | 1.90E-34 |
| comp25616_c0 | NA | -11.16 | 1.23E-2 |
| comp49586_c0 | valyl-tRNA synthetase | -11.14 | 8.83E-33 |
| comp56919_c0 | protein transport protein SEC61 subunit alpha | -11.08 | 6.11E-29 |
| comp47597_c0 | NA | -11.08 | 1.27E-2 |
| comp54131_c1 | NA | -10.99 | 1.32E-2 |
| comp48377_c0 | saposin | -10.98 | 7.674E-4 |
| comp43046_c0 | NA | -10.98 | 1.33E-2 |

^a^ Fold change was calculated as the number of reads per kilo bases per million (RPKM) of the midgut sample of *C. medinalis* treated with Cry1C divided by the RPKM of the midgut sample of *C. medinalis* treated without Cry1C.
