# Supplementary material for: Midgut transcriptomal response of the rice leaffolder, Cnaphalocrocis medinalis (Guenée) to Cry1C toxin
Source: PLoS One. 2018 Jan 23;13(1):e0191686. doi: 10.1371/journal.pone.0191686 (PMC5779695; doi:10.1371/journal.pone.0191686)
Supplement: S4 Table — (DOCX) [file pone.0191686.s007.docx]

**S4 Table**

Carboxylesterase genes differently expressed in *Cnaphalocrocis medinalis* larvae treated with Cry1C toxin.

| Gene ID | Annotation | Treated vs Control | | |
| --- | --- | --- | --- | --- |
|  |  | Log2FC^a^ | FDR | regulated |
| comp54912_c0 | carboxylesterase | 2.63 | 1.38E-40 | up |
| comp58070_c0 | carboxylesterase | 1.21 | 2.16E-26 | up |
| comp56982_c1 | carboxylesterase | 2.58 | 2.58E-14 | up |
| comp56049_c0 | carboxylesterase | 1.91 | 6.06E-13 | up |
| comp62636_c0 | carboxylesterase | 1.06 | 1.30E-10 | up |
| comp60068_c0 | carboxylesterase | 1.15 | 1.71E-07 | up |
| comp52634_c0 | carboxylesterase | 2.50 | 1.08E-05 | up |
| comp895940_c0 | carboxylesterase | 3.89 | 4.19E-03 | up |
| comp52546_c0 | carboxylesterase | 1.72 | 1.14E-02 | up |
| comp47981_c0 | carboxylesterase | 1.56 | 1.61E-02 | up |
| comp57757_c0 | carboxylesterase type B | 1.17 | 3.28E-06 | up |
| comp56544_c0 | carboxylesterase | -1.28 | 8.76E-12 | down |
| comp55426_c1 | carboxylesterase | -1.99 | 1.74E-05 | down |

^a^ Fold change was calculated as the number of reads per kilo bases per million (RPKM) of the midgut sample of *C. medinalis* treated with Cry1C divided by the RPKM of the midgut sample of *C. medinalis* treated without Cry1C.
